# Gemcitabine as maintenance treatment of diffuse pleural mesothelioma: randomized phase II study

**DOI:** 10.1186/s12885-026-15836-3

**Published:** 2026-03-26

**Authors:** Mohamed Emam Sobeih, Maha Helal, Maha Yahia, Ola Khorshid

**Affiliations:** 1 Medical Oncology Department, National Cancer Institute Cairo University, Giza, Egypt; 2 Radiology Department, National Cancer Institute Cairo University, Giza, Egypt

**Keywords:** Malignant Pleural Mesothelioma, Gemcitabine, Maintenance Therapy, Platinum-Based Chemotherapy, Best Supportive Care

## Abstract

**Background:**

Diffuse Pleural Mesothelioma (DPM) is an aggressive cancer with limited treatment options and a poor prognosis. Maintenance therapy has emerged as a potential strategy to delay disease progression following first-line chemotherapy. This study evaluates the efficacy and safety of gemcitabine maintenance therapy compared to best supportive care (BSC) in patients with unresectable DPM.

**Methods:**

This single-center, randomized, phase II, open-label trial included 64 patients with unresectable DPM who achieved a complete/partial response or stable disease after first-line platinum-based chemotherapy. They were randomized 1:1 to receive gemcitabine maintenance therapy (GEM group, *n* = 32) or BSC (BSC group, *n* = 32). Progression-free survival (PFS) was the primary endpoint, while overall survival (OS) and toxicity were secondary endpoints.

**Results:**

Gemcitabine maintenance therapy significantly improved PFS (median: 6.2 months vs. 2.8 months; HR: 4.33, 95% CI: 2.48–7.56, *p* < 0.001). However, no significant difference in OS was observed between the GEM and BSC groups (*p* = 0.155). Performance status (PS) at randomization and histological type were significant prognostic factors for survival. The GEM group experienced higher rates of hematological toxicities, while the BSC group reported more dyspnea, fatigue, and chest pain. No grade 3 or 4 toxicities were observed.

**Conclusion:**

Gemcitabine maintenance therapy effectively delays disease progression in patients with unresectable DPM, with a manageable toxicity profile. However, its impact on OS is limited. Performance status and histological type remain critical factors in predicting treatment outcomes.

**Trial Registration:**

ClinicalTrials.gov (ID NCT07411144, Date 1322026).

## Introduction

Diffuse pleural mesothelioma (DPM) is an exceedingly aggressive neoplasm that has emerged as a global health concern owing to its dismal prognosis and rising incidence rates. In 2020, 30,000 individuals were diagnosed with mesothelioma globally. The prevalence is greater in males compared to females and escalates with age; the median age at diagnosis is 76 years. The highest prevalence is observed in nations with the most extensive historical asbestos utilization, including the Netherlands, the United Kingdom, and Australia [1]. In Egypt, there were 375 newly diagnosed cases of mesothelioma in 2022, accounting for 0.35% of all cancer cases. The number of deaths attributed to mesothelioma during the same period was 335 [2].

DPM is typically resistant to local treatment and often advances quickly, leading to a median overall survival (OS) of 8–14 months for advanced cases [3]. Most patients with DPM are unsuitable for radical surgery due to extensive pleural metastases. Even in patients with resectable PM, the MARS 2 trial showed worse OS with extended pleurectomy decortication compared to chemotherapy alone [4]. In patients eligible for systemic treatment, the cisplatin and pemetrexed doublet combination yielded a survival advantage of 3 months compared to cisplatin alone [5]. The addition of bevacizumab to this doublet therapy can yield an approximate survival advantage of three months [3].

The introduction of immune checkpoint inhibitors represents a significant advancement in the treatment of diffuse pleural mesothelioma (DPM), especially for patients in advanced disease stages. Combination treatments, such as nivolumab and ipilimumab, have notably improved survival and patient outcomes [6]. The CheckMate 743 trial demonstrated long-term survival benefit over chemotherapy after a 3-year follow-up [7].

Unfortunately, nearly all patients who received first-line treatment experience a recurrence of the disease over time. The restricted efficacy of therapies underscores the need for further research and innovative strategies to improve patient outcomes. The idea of maintenance therapy has arisen as a therapeutic strategy designed to preserve a clinically favorable condition following successful first-line chemotherapy. For individuals qualified for supplementary therapies, many alternatives may be contemplated, including intravenous vinorelbine, gemcitabine, and pemetrexed retreatment [8]. Gemcitabine is an anticancer nucleoside that is an analogue of deoxycytidine and exhibits strong antitumor efficacy against a range of solid cancers. It acts by inhibiting DNA synthesis [9].

Therefore, in this study, we raised the question: Can the incorporation of additional systemic medication improve progression-free or overall survival rates? The study evaluated the efficacy of gemcitabine maintenance therapy after first-line chemotherapy treatment versus best supportive care only for patients with unresectable malignant pleural mesothelioma.

## Patients and methods

This randomized, phase II, open-label parallel trial was conducted at the National Cancer Institute (NCI), Cairo University, in the period from March 2020 to 2023. The study is compatible with the guidelines of good clinical practice and the principles of the Declaration of Helsinki and adheres to CONSORT guidelines. The study was approved by the Institutional Review Board of the NCI and registered in ClinicalTrials.gov (ID: NCT07411144). Each participant provided a written informed consent before enrollment after a complete explanation of the study protocol and its possible drawbacks and complications.

The study included patients with histologically proven unresectable diffuse pleural mesothelioma (DPM) who had a complete or partial response or stable disease after four to six cycles of first-line platinum-based chemotherapy (CTH), according to modified Response Evaluation Criteria in Solid Tumors (mRECIST) criteria. The patients were included if the last CTH dose was administered within 60 days before randomization. Inclusion criteria included age > 18 years, Eastern Cooperative Oncology Group (ECOG) performance status 0–2, life expectancy of at least 12 weeks, adequate bone marrow, and acceptable liver and renal functions.

The exclusion criteria were a history of extra-pleural pneumonectomy, active brain or leptomeningeal metastases, weight loss > 10% within 6 weeks before enrollment, clinically significant ascites, or known intolerance to gemcitabine. Moreover, patients were excluded if they received non-palliative radiotherapy within 3 weeks before the study treatment initiation.

### Study procedure

Baseline assessment was performed before randomization, including echocardiography, CT, or MRI of the chest and upper abdomen. Initial brain MRI or CT scans and bone scans were obtained if clinically indicated. Laboratory tests, including complete blood count and serum chemistry, were to be performed within 7 days before the first dose of the study treatment. Absence of progression was judged based on clinical and radiological criteria according to the mRECIST for pleural mesothelioma.

Randomization of the participants was performed using computerized sequence generation (http://www.randomizer.org/). The study participants were randomly assigned in a 1:1 ratio to receive either gemcitabine 1000 mg/m^2^ intravenously on days 2 and 8 of a 21-day-long cycle in addition to best supportive care (GEM Group, *n* = 32) or best supportive care only (BSC Group, *n* = 32).

Supportive care visits were arranged every 3 weeks. The supportive schedule included psychosocial treatment, managing other needs, and providing appropriate pain and pleural effusion management. Pleural fluid drainage or palliative radiotherapy for pain management were provided as needed.

The patients were treated until disease progression, intolerable toxicity, or patient or physician decision to discontinue. Dose reduction (75% of the full dose) was done if the absolute neutrophil count (ANC) became 500 to < 100 × 10^6^/L or the platelet count of 5000 to < 1000 × 10^6^/L.

Patients were monitored for disease progression according to mRECIST criteria by thoracoabdominal CT every 8 weeks, regardless of dose administration delays, treatment interruptions, or early treatment discontinuation. After treatment discontinuation, all participants were followed up every 3 months until death. In case of discontinuation without progression, patients were followed every 8 weeks to register the disease progression.

### Outcomes

The primary outcome measure was progression-free survival (PFS). The secondary outcomes included objective response rate and the factors associated with disease progression and overall survival (OS). PFS was calculated from the date of randomization until the date of first documented progression according to mRECIST or death or end of follow-up. OS was calculated from the date of randomization to the date of death or end of follow-up. The adverse events, regardless of cause, were assessed using the Common Terminology Criteria for Adverse Events (CTCAE), version 4.0, throughout the study period.

### Sample size estimation

Based on median survival data from a previous study [10], a minimum sample size of 28 patients per arm was needed at an alpha error of 0.05 and a power of 80%. A 15% increase in the sample size was added to compensate for possible losses to follow up. Thus, the final sample size was 32 patients per arm (total 64 patients). The sample size was calculated using PS: Power and Size Calculation, version 3.1.2.

### Statistical analysis

Statistical analysis was done using IBM© SPSS© Statistics version 26 (IBM© Corp., Armonk, NY, USA). Efficacy and safety were analyzed in all randomly assigned patients. Numerical data are expressed as mean and standard deviation (SD), median, or range as appropriate according to the distribution. Qualitative data are expressed as frequency and percentage. Chi-square test or Fisher’s exact test was used to assess the relationships between qualitative variables, and t-test or Mann-Whitney test was used to compare continuous variables between independent groups, as appropriate. Progression-free and overall survival analyses were performed using Kaplan-Meier curves. Comparison of survival data between the two arms was done using the log-rank test. A Cox proportional hazards model was constructed for multivariate analysis of factors affecting survival. A p-value < 0.05 was considered significant, and all tests were two-tailed.

## Results

**Fig. 1:**
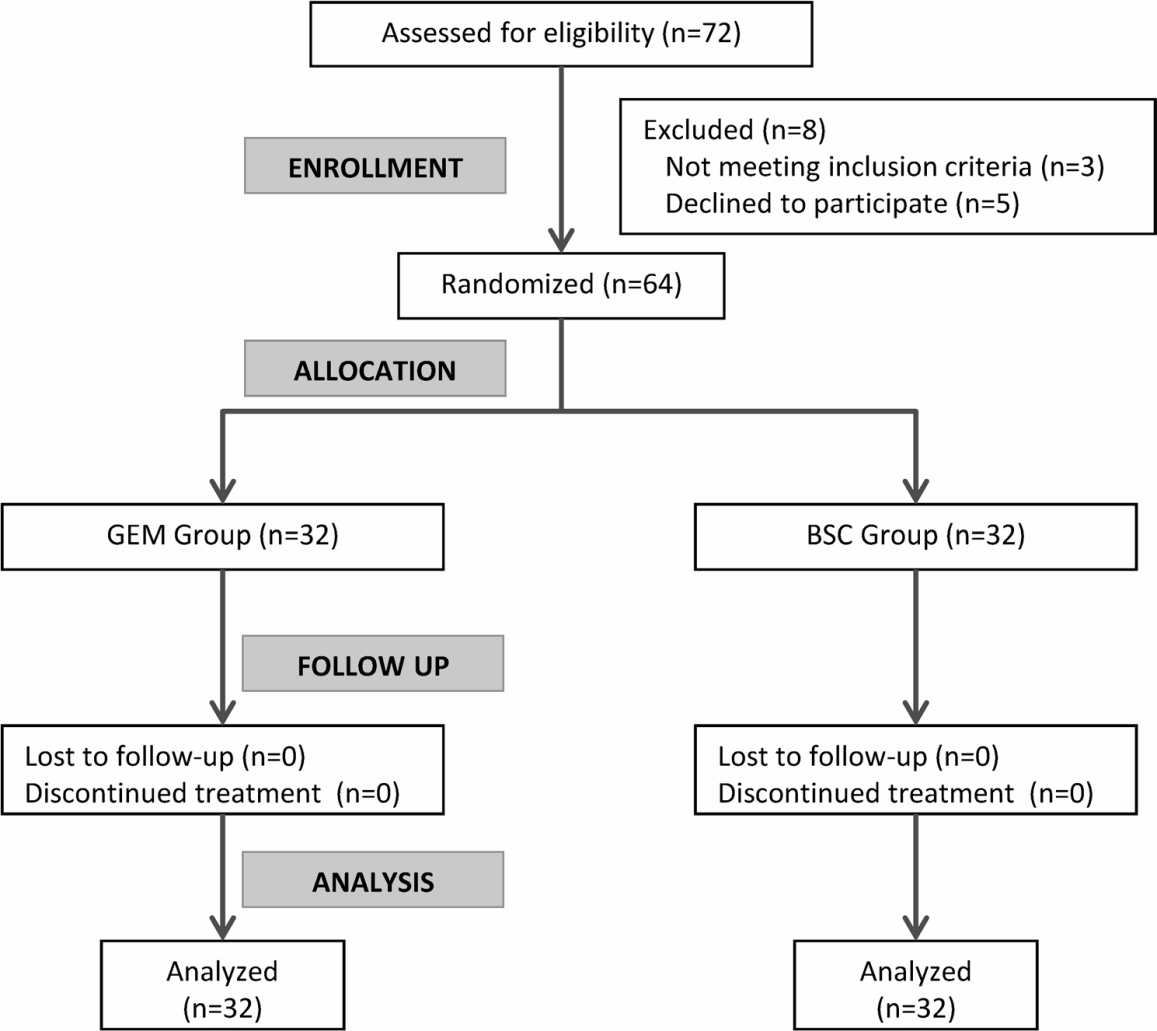
CONSORT flow diagram

Of the 72 patients evaluated, 3 were not eligible according to the exclusion criteria, and 5 patients refused to participate in the study (Fig. 1). Table 1 shows significant differences between the two groups in body mass index (*p* = 0.026), PS at randomization (*p* = 0.035), and total leucocytic count (*p* = 0.001). Other baseline characteristics were comparable between groups. The median follow-up duration was 15.4 months (range: 3–35 months). No patients were lost to follow-up. The baseline differences were considered in survival analysis.

**Table 1:** Baseline characteristics of the two studied groups

	GEM Group(*n* = 32)	BSC Group(*n* = 32)	*p*-value
Age (years)	55.0 ± 9.00	56.7 ± 11.8	0.538
< 60	22 (68.8%)	18 (56.3%)	0.302
≥ 60	10 (31.3%)	14 (43.8%)	
Sex (Male/Female)	21/11	16/16	0.206
Body mass index (kg/m^2^)	26.21 ± 5.67	29.84 ± 6.99	0.026
Body surface area (m^2^)	1.7 ± 0.2	1.8 ± 0.2	0.051
Smoking	12 (37.5%)	13 (40.6%)	0.798
Diabetes mellitus	7 (21.9%)	5 (15.6%)	0.522
Hypertension	5 (15.6%)	7 (21.9%)	0.522
ECOG PS at randomization			0.035
PS I	25 (78.1%)	17 (53.1%)	
PS II	7 (21.9%)	15 (46.9%)	
Histology			1.000
Epithelioid	28 (87.5%)	27 (84.4%)	
Non-epithelioid	4 (12.5%)	5 (15.6%)	
Stage			0.351
Stage III	5 (15.6%)	8 (25.0%)	
Stage IV	27 (84.4%)	24 (75.0%)	
Number of Cycles of 1st line Chemotherapy			0.424
4–5 cycles	9 (28.1%)	12 (37.5%)	
6 cycles	23 (71.9%)	20 (62.5%)	
Response 1st line Chemotherapy			0.209
Regressive	17 (53.1%)	12 (37.5%)	
Stationary	15 (46.9%)	20 (62.5%)	
Hemoglobin (g/dl)	10.6 ± 1.4	11.1 ± 1.5	0.198
Total Leucocytic Count (x10^9^/L)	6.2 ± 2.4	9.2 ± 4.6	0.001
Platelet count (x10^9^/L)	291 (100–668)	330 (198–960)	0.053

Data are presented as Mean (SD) or number (%)

*ECOG PS* Eastern Cooperative Oncology Group Performance Status

Most tumors in the two groups were of the epithelioid type (*p* = 1.000). Most patients presented with stage IV disease. Six cycles of CTH were used in most patients in the induction phase. There was no significant difference in response to first-line CTH between the two groups (*p* = 0.209). Gemcitabine maintenance therapy was associated with significantly fewer patients with disease progression (*p* < 0.001). Second-line chemotherapy was administered to 22 patients in each group, with no significant difference in response between the two groups (*p* = 0.092) (Table 2).

**Table 2:** Response to maintenance therapy and second-line chemotherapy in the two studied groups

	GEM Group (*n* = 32)	BSC Group (*n* = 32)	*p*-value
Response to maintenance			< 0.001
Regressive (Complete or partial response)	10 (31.3%)	1 (3.1%)	
Stable Disease	17 (53.1%)	7 (21.9%)	
Progressive	5 (15.6%)	24 (75.0%)	
Response to 2nd line*			0.092
Regressive	4 (18.2%)	1 (4.5%)	
Stable Disease	4 (18.2%)	11 (50.0%)	
Progressive	14 (63.6%)	10 (45.5%)	

Data are presented as number (%)

* Each group involved 22 patients

### Survival analysis

**Fig. 2:**
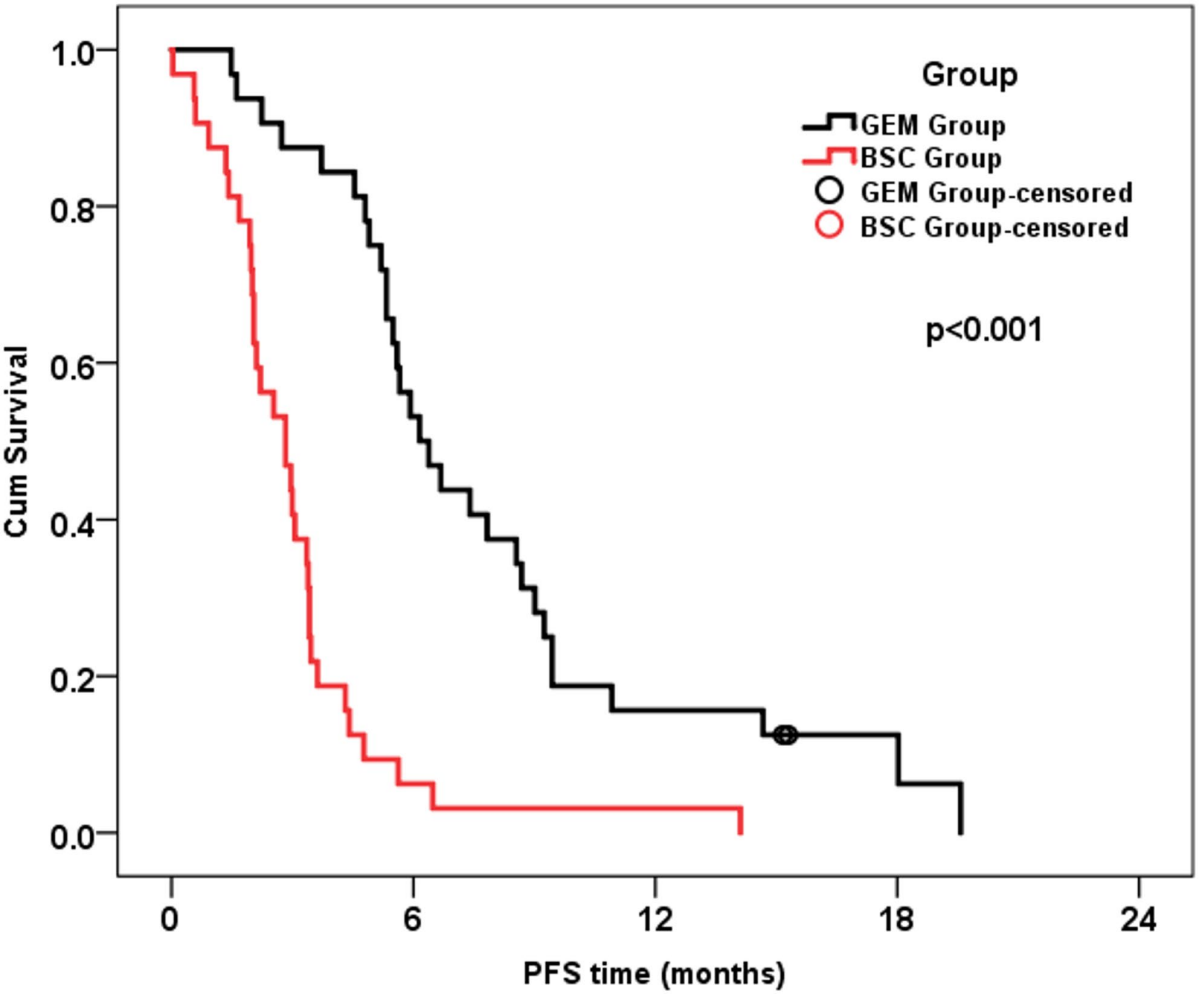
Progression-free survival of the two studied groups

The median time to disease progression was 4.4 months (range: 0.03–19.6). The cumulative PFS of the whole group at 6 months was 29.7%. The median PFS was 4.3 months (95%CI: 3.0-5.7 months). PFS was significantly better in the GEM group compared to the BSC group (Hazard ratio [HR] for BSC alone: 4.33, 95% CI: 2.48–7.56, Fig. 2). Performance status at randomization significantly affected PFS (HR: 1.83, 95%CI: 1.06–3.16), where patients with PS II have worse PFS than those with PS I (*p* = 0.028). PFS was not affected by other patients’ and tumor characteristics (Table 3). On multivariate analysis, gemcitabine maintenance therapy was the only independent factor affecting PFS (HR: 4.19, 95%CI: 2.37–7.40).

**Table 3:** Progression-free survival and its relation to prognostic factors

	Events/n	Cumulative survival at 6 months (%)	Median survival (months)	HR (95% CI)	*p*-value
Whole group	62/64	29.7	4.3 (3.0-5.7)		
Group				4.33 (2.48–7.56)	**< **0.001
GEM*	30/32	53.1	6.2 (4.7–7.6)		
BSC	32/32	6.3	2.8 (1.8–3.9)		
Age groups				1.21 (0.71–2.05)	0.475
< 60 years*	39/40	37.5	4.5 (3.2–5.9)		
≥ 60 years	23/24	20.8	3.4 (2.8–3.9)		
Sex				1.01 (0.60–1.68)	0.984
Male*	36/37	29.7	4.8 (2.9–6.7)		
Female	26/27	29.6	3.6 (3.0-4.2)		
PS at randomization				1.83 (1.06–3.16)	0.028
PS I*	41/42	40.5	5.5 (4.6–6.4)		
PS II	21/22	9.1	3.1 (2.4–3.7)		
NLR				1.32 (0.79–2.21)	0.278
≤ 0.8	28/29	37.9	5.3 (3.4–7.2)		
> 0.8	24/35	22.9	3.4 (2.3–4.5)		
Histology				1.55 (0.76–3.17)	0.226
Epithelioid*	53/55	30.9	4.5 (3.2–5.9)		
Non-Epithelioid	9/9	22.2	2.1 (1.6–2.6)		
Stage				0.85 (0.46–1.58)	0.605
Stage III*	13/13	23.1	2.5 (0.0-5.8)		
Stage IV	49/51	31.4	4.4 (3.0-5.9)		
Response to 1st line CTH				1.20 (0.72–2.01)	0.475
Regressive*	28/29	31.0	4.4 (1.2–7.6)		
Stationary	34/35	28.6	4.3 (2.8–5.8)		
No. of Cycles of 1st line CTH				1.25 (0.73–2.15)	0.411
4–5 cycles	20/21	23.8	3.4 (1.2–5.6)		
6 cycles*	42/43	32.6	4.4 (2.6–6.2)		

*HR* Hazard ratio, *CI* Confidence interval

* Reference group for HR calculation

The median follow-up period till death was 15.4 months (range: 3.0-35.2). The cumulative overall survival (OS) of the whole group at 18 months was 52.2%. The median OS was 19.5 months (range: 13.2–25.7 months). The use of gemcitabine maintenance therapy did not affect OS (*p* = 0.155). Patients with PS II carried a higher risk of worse OS (HR: 2.67, 95%CI: 1.40–5.08). Non-epithelioid tumors have significantly worse OS (HR: 3.85, 95%CI: 1.70–8.74). Also, 4–5 CTH cycles were associated with worse OS than 6 cycles (HR: 2.52, 95%CI: 1.27–5.01) (Table 4).

**Table 4:** Overall survival and its relation to prognostic factors

	Events/n	Cumulative survival at 6 months (%)	Median survival (months)	HR (95% CI)	*p*-value
Whole group	39/64	52.2	19.5 (13.2–25.7)		
Group				1.58 (0.84-3.00)	0.155
GEM*	16/32	61.7	23.3 (15.7–30.8)		
BSC	23/32	42.9	13.4 (9.5–17.3)		
Age groups				1.49 (0.79–2.84)	0.217
< 60 years*	23/40	58.7	19.6 (13.1–26.0)		
≥ 60 years	16/24	41.7	12.4 (10.7–14.1)		
Gender				1.25 (0.67–2.36)	0.484
Male*	21/37	56.1	19.5 (11.5–27.4)		
Female	18/27	46.9	15.7 (6.5–24.9)		
PS at randomization				2.67 (1.40–5.08)	0.002
PS I*	22/42	65.7	23.3 (17.4–29.1)		
PS II	17/22	27.3	10.9 (8.3–13.5)		
Histology				3.85 (1.70–8.74)	0.001
Epithelioid*	31/55	59.3	21.6 (17.3–25.9)		
Non-Epithelioid	8/9	0.0	10.9 (7.6–14.3)		
Stage				1.99 (0.83–4.75)	0.116
Stage III*	6/13	84.6	23.2 (20.4–25.9)		
Stage IV	33/51	44.0	15.4 (11.9–18.8)		
Response to 1st line CTH				1.14 (0.60–2.16)	0.684
Regressive*	17/29	53.0	18.6 (9.9–27.3)		
Stationary	22/35	51.1	19.5 (9.8–29.1)		
No. of Cycles of 1st line CTH				2.52 (1.27–5.01)	0.006
4–5 cycles	15/21	31.7	12.4 (8.5–16.3)		
6 cycles*	24/43	62.1	23.2 (17.8–28.5)		

*HR* Hazard ratio, *CI* Confidence interval

* Reference group for HR calculation

Disease progression was not affected by age, sex, histological type, stage, or type of first-line CTH (Table 5). PS II at randomization was associated with a higher risk of disease progression (Risk ratio [RR]: 2.05, 95%CI: 1.22–3.42). A lower proportion of patients who received six cycles of CTH developed progression compared to those who received 4 or 5 cycles. However, the difference was not statistically significant (*p* = 0.062).

**Table 5:** Factors associated with disease progression

	Regressive/Stable disease (*n* = 35)	Progressive (*n* = 29)	Risk Ratio (95% CI)	*p*-value
Age (years)	54.9 ± 8.9	57.0 ± 12.2		0.425
Sex			0.90 (0.52–1.54)	0.697
Male*	21 (56.8%)	16 (43.2%)		
Female	14 (51.9%)	13 (48.1%)		
PS at randomization			2.05 (1.22–3.42)	0.006
PS I	28 (66.7%)	14 (33.3%)		
PS II*	7 (31.8%)	15 (68.2%)		
Histology			0.98 (0.45–2.15)	0.955
Epithelioid	30 (54.5%)	25 (45.5%)		
Non-Epithelioid*	5 (55.6%)	4 (44.4%)		
Stage			0.80 (0.44–1.45)	0.489
Stage III	6 (46.2%)	7 (53.8%)		
Stage IV*	29 (56.9%)	22 (43.1%)		
First line CTH				
Number of Cycles			1.66 (1.00-2.78)	0.062
4–5 cycles*	8 (38.1%)	13 (61.9%)		
6 cycles	27 (62.8%)	16 (37.2%)		
Response			0.89 (0.52–1.52)	0.665
Regressive	15 (51.7%)	14 (48.3%)		
Stationary*	20 (57.1%)	15 (42.9%)		

Data are presented as mean (SD) or number (%)

*PS* Performance status, *CI* Confidence interval

* Reference group for risk estimation

Dyspnea, fatigue, and chest pain were significantly more common in patients of the BSC group, while anemia, leucopenia, and nausea were more common in the GEM group (Table 6). There is no grade 3 or 4 toxicity in both groups.

**Table 6:** Frequency of toxicity manifestations encountered in the two studied groups

	GEM Group (*n* = 32)	BSC Group (*n* = 32)	*p*-value
Dyspnea	4 (12.5%)	30 (93.8%)	< 0.001
Fatigue	2 (6.3%)	30 (93.8%)	< 0.001
Chest pain	7 (21.9%)	30 (93.8%)	< 0.001
Anemia	28 (87.5%)	0 (0.0%)	< 0.001
Leucopenia	19 (59.4%)	0 (0.0%)	< 0.001
Nausea	12 (37.5%)	0 (0.0%)	< 0.001
Renal	4 (12.5%)	0 (0.0%)	*
Diarrhea	3 (9.4%)	0 (0.0%)	*
Peripheral neuropathy	3 (9.4%)	1 (3.1%)	*
Edema	2 (6.3%)	0 (0.0%)	*
Fever	1 (3.1%)	0 (0.0%)	*
Bony pain	1 (3.1%)	0 (0.0%)	*
Hypotension	1 (3.1%)	0 (0.0%)	*
Anorexia	1 (3.1%)	0 (0.0%)	*
Constipation	1 (3.1%)	0 (0.0%)	*
Pain	0 (0.0%)	2 (6.3%)	*

Data are presented as number (%)

* No *p*-value because of small numbers in subgroups

## Discussion

This study offers valuable insights into the effectiveness and safety of gemcitabine switch maintenance therapy for patients with advanced pleural mesothelioma, focusing on PFS, OS, and toxicity profile. Gemcitabine maintenance significantly improved PFS compared to BSC alone, increasing the median from 2.8 to 6.2 months (HR: 4.33, 95% CI: 2.48–7.56). Multivariate analysis identified gemcitabine maintenance as the only independent factor influencing PFS (HR: 4.19, 95% CI: 2.37–7.40). These results highlight gemcitabine’s potential to delay disease progression and support continued investigation into its use as maintenance therapy.

While GEM improved PFS, it did not significantly impact OS (*p* = 0.155). Meanwhile, OS was worse in patients with PS II (HR: 2.67, *p* = 0.002), non-epithelioid tumors (HR: 3.85, *p* = 0.001), and those receiving fewer than six cycles of first-line chemotherapy (4–5 cycles) compared to six cycles (HR: 2.52, *p* = 0.006).

Maintenance therapy is an effective approach in treating solid tumors and is known to extend PFS in many cancer types [11–17]. However, the significance of maintenance therapy for mesothelioma remains unclear.

Current research on maintenance therapy for DPM is limited, with few studies exploring alternative options. Switch-maintenance with non-cross-resistant drugs like thalidomide has been unsuccessful [18]. A randomized trial found that defactinib maintenance after first-line chemotherapy did not improve PFS or OS in Merlin-low DPM patients; thus, its use is not recommended [19]. The DENIM trial is an ongoing multicenter, randomized, phase II/III study of dendritic cells loaded with allogeneic tumor cell lysate as maintenance therapy for mesothelioma after chemotherapy [20]. Continuation maintenance with pemetrexed after initial pemetrexed and platinum chemotherapy does not enhance PFS in patients with DPM [21].

A more recent study reported significant prolongation of PFS with a controllable toxicity profile with switch-maintenance gemcitabine after standard first-line platinum and pemetrexed therapy [22]. The current study is another randomized trial supporting this new treatment strategy for malignant mesothelioma, following the NVALT19 study [22] and Karam et al. [23]. In agreement with the current research, these two studies demonstrated prolonged PFS but not OS compared with BSC.

CALGB 30,901 [21], a randomized phase 2 trial, examined the efficacy of maintenance pemetrexed versus observation following initial treatment in a cohort of 49 patients, most of whom received carboplatin and pemetrexed as first-line therapy. The results indicated no statistically significant enhancement in progression-free survival (HR 0.99, *p* = 0.973) or overall survival (HR 0.86, *p* = 0.674), while pemetrexed maintained a manageable toxicity profile.

Gemcitabine was examined in adults with DPM who experienced progression during or following first-line treatment with pemetrexed and platinum in a multicenter, randomized, double-blind, phase 2 trial (RAMES trial). They were randomly assigned to receive gemcitabine plus placebo or gemcitabine plus ramucirumab. In this study, adding ramucirumab to gemcitabine resulted in a longer OS (*p* = 0.028). Grade 3–4 treatment-related adverse events were reported in 44% of patients in the gemcitabine plus ramucirumab group and 30% in the gemcitabine plus placebo group. The most common events were neutropenia and hypertension [24].

Other treatment modalities were investigated in mesothelioma patients progressing during or after first-line therapy. A recent retrospective study provided real-world clinical evidence on the use of Immune checkpoint inhibitors (ICIs) for advanced or relapsed disease [25]. The PROMISE-meso trial compared pembrolizumab and single-agent chemotherapy in relapsed DPM progressing after platinum-based chemotherapy. After a median follow-up of 17.5 months, no significant difference was found in PFS or OS [26]. The CONFIRM trial, a multicenter, placebo-controlled, double-blind, parallel-group, randomized phase 3 trial, found no significant difference in OS between nivolumab and placebo in adults with radiological evidence of disease progression after platinum-based chemotherapy. The most frequent grade 3 adverse events were diarrhea and infusion-related reactions [27].

Gemcitabine maintenance therapy has been studied for various solid tumor types, including squamous cell lung cancer [28], unresectable non-small-cell lung cancer (NSCLC) [29], metastatic urothelial cancers [30, 31], and metastatic breast cancer [32]. It has shown potential benefits in improving PFS and OS in patients with good PS and objective response to platinum-gemcitabine chemotherapy. However, the potential response rate is low and insufficient for patients with stable disease following first-line chemotherapy [33]. A randomized phase III study found that maintenance with gemcitabine in advanced NSCLC produced a longer time to progression compared to best supportive care, but did not improve OS [34]. A prospective observational study evaluated the efficacy and safety of the combination of Gemcitabine plus nab-Paclitaxel, followed by maintenance Gemcitabine in older adults with locally advanced or metastatic pancreatic cancer [35].

Performance status at randomization emerged as a critical prognostic factor for both PFS and OS in this study. Moreover, PS significantly impacted the response to maintenance therapy. Patients with PS I had significantly better outcomes compared to those with PS II. ​ This finding highlights the importance of baseline functional status in predicting treatment efficacy and survival. Histological type also influenced OS, with non-epithelioid tumors associated with worse outcomes. ​ These results emphasize the need for personalized treatment strategies based on patient and tumor characteristics.

In patients with lung cancer, PS has been shown to be a prognostic indicator of survival advantage for maintenance therapy [34]. It was shown that PFS was significantly extended in patients with a PS of 0 compared to those with a PS of 1, while OS benefits were not significantly different [36, 37]. Furthermore, no substantial survival advantage in PFS and OS was noted in patients with a PS of 2, even when administered well-tolerated medicines [33]. Lack of PFS advantage in the study of Belani and colleagues [38] is likely attributable to the elevated percentage of patients with a PS of 2 (64%) at baseline. Consequently, PS should be evaluated prior to commencing maintenance therapy.

The toxicity profile differed significantly between the GEM and BSC groups. ​The GEM group had higher rates of hematological toxicities such as anemia (87.5%), leucopenia (59.4%), and nausea (37.5%). ​The BSC group experienced significantly more dyspnea (93.8%), fatigue (93.8%), and chest pain (93.8%) (*p* < 0.001). ​Importantly, no grade 3 or 4 toxicities were reported in either group, indicating that gemcitabine maintenance therapy is relatively safe and well-tolerated. ​However, the higher incidence of hematological toxicities in the GEM group warrants careful monitoring during treatment. ​Gemcitabine is a generally well-tolerated chemotherapeutic agent, with hematotoxicity being the most important major side effect [39].

### Clinical implications

The study demonstrates that gemcitabine maintenance therapy can significantly delay disease progression in patients with unresectable DPM, offering a viable option for maintenance treatment. ​ However, its impact on OS is limited, suggesting that additional therapeutic strategies are needed to improve long-term survival. ​ The findings also highlight the importance of optimizing first-line chemotherapy regimens and ensuring patients receive the full course of treatment to maximize survival benefits. Patients with a better PS appear to be more likely to benefit from gemcitabine maintenance.

### Limitations

The study has some limitations. The relatively small sample size may limit the generalizability of the findings. Being a single-center study is also a considerable limitation. However, the NCI is the main referral center for cancer in Egypt, and consequently, the included patients can be a representative sample of the country’s population. Another limitation of this study is the lack of a validated quality-of-life (QoL) questionnaire, which prevents assessment of the patients’ subjective well-being after treatment beyond objective clinical indicators. Additionally, the lack of significant OS improvement with gemcitabine maintenance therapy raises questions about its long-term benefits. Further research is needed to explore combination therapies or alternative maintenance strategies that may enhance survival outcomes.

## Conclusion

Gemcitabine maintenance therapy is effective in improving PFS and is associated with manageable toxicity. ​ However, its impact on OS is limited, and other factors such as performance status, histological type, and treatment intensity play a more significant role in determining survival outcomes. Future studies should focus on optimizing maintenance strategies and exploring novel therapeutic approaches to improve long-term survival in patients with advanced malignancies.

## Data Availability

The dataset used and analyzed during the current study is available from the corresponding author on reasonable request.
